# Posterior Reversible Encephalopathy Syndrome Associated With a Pontine Lacunar Infarct in a Severely Hypertensive Patient: A Case Report

**DOI:** 10.7759/cureus.73995

**Published:** 2024-11-19

**Authors:** Paige Conrad, Jennifer John, Andrea De Lemos, Alejandro Biglione

**Affiliations:** 1 Internal Medicine, Nova Southeastern University Dr. Kiran C. Patel College of Osteopathic Medicine, Davie, USA; 2 Internal Medicine, Wellington Regional Medical Center, Wellington, USA

**Keywords:** brainstem, hypertension, pontine infarct, reversible encephalopathy, vasogenic brain edema

## Abstract

Posterior reversible encephalopathy syndrome (PRES) is a neurologic condition defined by symptoms and imaging findings secondary to vasogenic edema in the brain. Even though not all hypertensive individuals will progress to PRES, high blood pressure is the most frequent risk factor associated with the condition. The pathophysiology of PRES is not clearly understood, but the most accepted proposed mechanism focuses on the brain's inability to regulate cerebral blood flow through constriction or dilation of vessels during extreme blood pressure. This case report is about a 38-year-old male patient who presented to the hospital complaining of a headache and was found to have severe hypertension associated with PRES and a pontine infarct. The epidemiology, etiology, pathophysiologic mechanism, clinical presentation, radiologic findings, management, and prognosis of PRES syndrome are discussed.

## Introduction

Posterior reversible encephalopathy syndrome (PRES) is a neurologic condition defined by symptoms and imaging findings secondary to vasogenic edema in the brain. It may occur in both sexes and at any age but is most frequently observed in middle-aged females [1]. In cases documented thus far, an estimated 70% of individuals who develop PRES have hypertension. The parietal and occipital regions of the cortex are most commonly affected in PRES, but isolated involvement of the brainstem has been documented in a few cases. In a study examining 124 confirmed cases of PRES, only 4% of cases involved infarction in the subcortical regions, such as the basal ganglia, cerebellum, thalami, and pons, resulting in infarction in these areas [2]. Another study examining 76 confirmed cases of PRES reported that 18% involved the brainstem [2]. The pons are one of the most susceptible regions of the brain to ischemia in PRES, and rapid diagnosis and treatment of a pontine infarct is essential to prevent irreversible damage [3]. This case report presents the case of a 38-year-old male patient with a past medical history of chronic kidney disease who presented to the hospital with complaints of a constant headache and was found to have PRES complicated by a pontine infarct.

## Case presentation

A 38-year-old male patient with a past medical history of chronic kidney disease presented to the hospital with complaints of a constant holocranial headache, which occurred on more than half of the days over the last two months. On the day of admission, the headache was located in the posterior occipital and nape of the neck region. He described the pain as throbbing in nature, moderate in intensity, and associated with new-onset dizziness and progressive numbness and tingling over the left side of his body, which prompted him to get evaluated. He had been taking Excedrin Tension, which mildly alleviated the pain and endorsed no known exacerbating factors. He denied any photophobia, phonophobia, chest pain, shortness of breath, nausea, vomiting, diarrhea, fever, syncope, altered speech, altered level of consciousness, seizures, changes in vision, unilateral weakness, ataxia, or changes in bowel or bladder control. 

The patient's medical history was significant for chronic kidney disease, but he had no pertinent surgical, social, or family history and was not taking any medications at home. The patient presented with a temperature of 36.9℃, a blood pressure (BP) of 245/171 mmHg, a pulse rate of 97 beats per minute, a respiratory rate (RR) of 20 breaths per minute, an oxygen saturation of 98% on room air, and a body mass index (BMI) of 29.95 kg/m^2^.

During the physical exam, the patient was awake, alert, and oriented to person, place, and time. No jugular venous distention was observed, and the heart rate and rhythm were regular, with no murmurs, rubs, or gallops. Lungs were clear to auscultation bilaterally, the abdomen was soft and nontender, and bowel sounds were present. No rashes, lesions, or edema were present. No cyanosis, clubbing, or gross deformities were present, and a normal range of motion was observed for all aspects of the musculoskeletal exam. The patient had fluent speech without dysarthria or compression, and expression was intact. Pupils were equal, round, and reactive to light and accommodation, and extraocular muscles were intact. The facial sensation was intact, with no facial droop. The tongue protruded at midline without deviation, the hearing was intact bilaterally, and a normal shoulder shrug was observed. On the motor exam, normal bulk and tone and 5/5 strength throughout were observed. No focal motor weakness was present. Sensitivity to light touch and pinprick touch bilaterally was intact, and deep tendon reflexes were 2+ bilaterally and symmetric. Finger-to-nose testing was intact bilaterally. Gait was not assessed. 

On admission, the patient's complete metabolic panel was ordered (Table 1), in addition to a complete blood count and general coagulation study (Tables 2-3). Cardiac lab values were also obtained (Table 4).

**Table 1:** Metabolic panel on admission BUN: Blood urea nitrogen; ALT: Alanine aminotransferase; AST: Aspartate aminotransferase; eGFR Cr: Estimated glomerular filtration rate of creatinine

	Initial Value	Normal Range
Sodium	134 mmol/L	135-148 mmol/L
Potassium	3.5 mmol/L	3.6-5.2 mmol/L
CO_2_	26 mEq/L	21-32 mEq/L
Chloride	98 mmol/L	95-110 mmol/L
Anion Gap	13.5 mmol/L	5-15 mmol/L
Albumin	4.3 gm/dL	3.4-5.0 gm/dL
BUN	32 mg/dL	7-18 mg/dL
Creatinine	2.82 mg/dL	0.70-1.30 mg/dL
Calcium	9.8 mg/dL	8.5-10.1 mg/dL
Glucose	99 mg/dL	74-106 mg/dL
ALT	24 Intl units/dL	12-78 Intl units/dL
AST	22 Intl_units/L	15-37 Intl_units/L
eGFR Cr	28 mL/min/1.73m^2^	≥ 60 mL/min/1.73m^2^
Alkaline phosphatase	90 Intl_units/L	45-117 Intl_units/L
Total Protein	8.3 gm/dL	6.4-8.2 gm/dL
Total Bilirubin	1.2 mg/dL	0.0-1.0 mg/dL

**Table 2:** General coagulation values on admission PT: Prothrombin time; INR: International normalized ratio; PTT: Partial thromboplastin time

	Initial Value	Normal Range
PT	11.4 seconds	9.7-11.7 seconds
INR	1.06	0.9-1.2
PTT	31.8 seconds	24-35 seconds

**Table 3:** Complete blood count laboratory values on admission WBC: White blood cells; RBC: Red blood cells; Hgb: Hemoglobin; HCT: Hematocrit; MCV: Mean corpuscular volume; MCH: Mean corpuscular hemoglobin; MCHC: Mean corpuscular hemoglobin concentration; RDW-CV: Red cell distribution width-coefficient of variation; RDW-SD: Red blood cell distribution width standard deviation

	Initial Value	Normal Range
WBC	5.17x10^3^/mcL	4.50 - 10.50 x 10^3^/mcL
RBC	6.32x10^6^/mcL	4.40 - 6.15 x 10^6^/mcL
Hgb	13.3 gm/dL	14-18 gm/dL
HCT	40.2%	40.0-54.0%
Platelets	221 x 10^3^/mcL	150 - 450 x 10^3^/mcL
MCV	63.6 Femtoliters	81.0 - 96.0 Femtoliters
MCH	21.0 pg	27.0 - 34.0 pg
MCHC	33.1 gm/dL	32-36 gm/dL
RDW-SV	36.7 Femtoliter	36.0-50.0 Femtoliter
RDW-CV	17.90%	11.00 -14.50%
Neutrophil %	64.4%	40-77%
Lymphocyte %	25.5%	24-44%
Monocyte %	8.1 %	0.0-15.0%
Eosinophil %	0.8%	0.0-10.0%
Basophil %	1.0%	0.0-2.0%
Immature Grans %	0.2%	0.0-5.0%
Neutrophil #	3.33 x10^3^/mcL	1.80-7.70 x 10^3^/mcL
Lymphocyte #	1.32 x 10^3^/mcL	0.80-2.80 x 10^3^/mcL
Monocyte #	0.42 x 10e3/mcL	0.20-1.00 x 10e3/mcL
Eosinophil #	0.04 x 10e3/mcL	0.00-0.60 x 10e3/mcL
Basophil #	0.05 x 10e3/mcL	0.00-0.30 x 10e3/mcL
Immature Grans #	0.01 x10e3/mcL	0.00-0.50 x 10e3/mcL

**Table 4:** Cardiac lab values on admission ProBNP: Pro-brain natriuretic peptide

	Initial Value	Normal Range
ProBNP	5902	0-169
Troponin	1361.5 ng/L	0.0-78.5 ng/L

**Figure 1:**
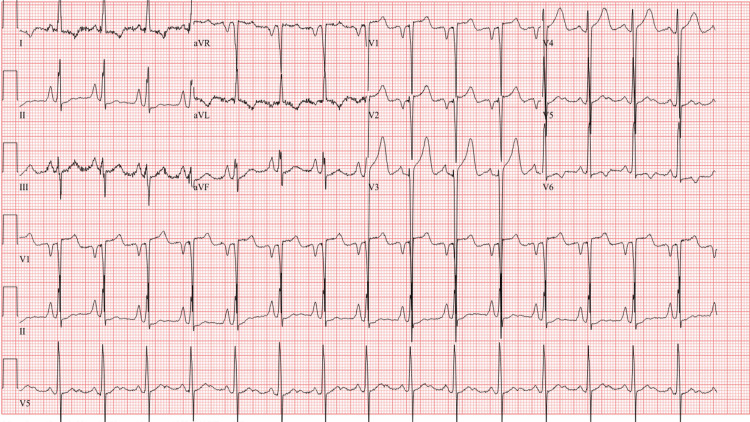
EKG showed normal sinus rhythm with left ventricular hypertrophy

**Figure 2:**
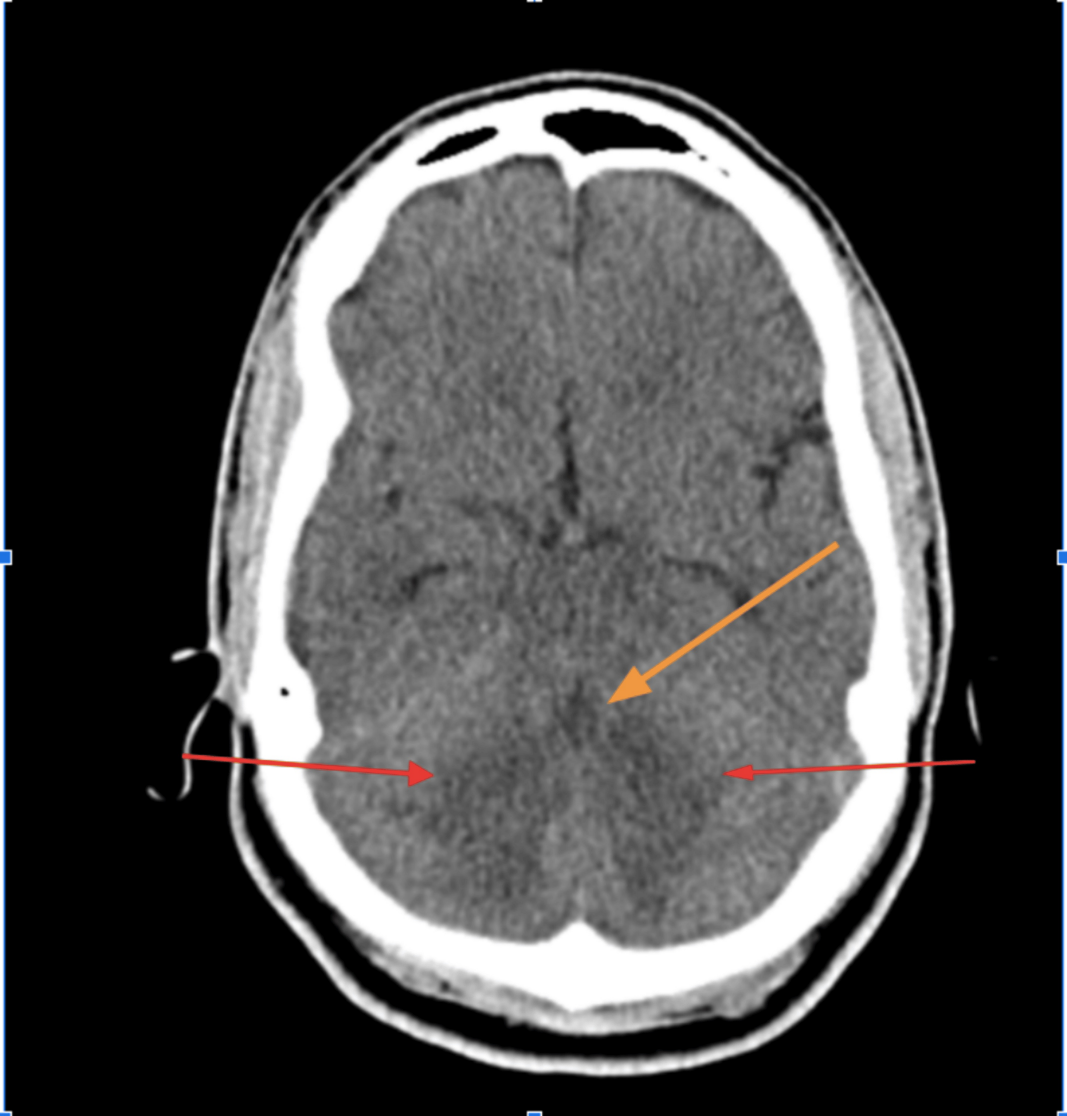
CT of the brain without contrast (axial view) The image shows a large hypodense area involving the cerebellum representing cerebellar edema (red arrows) and mild localized mass effect with partial effacement of the fourth ventricle shifted slightly to the right of midline (orange arrow). CT: computed tomography

**Figure 3:**
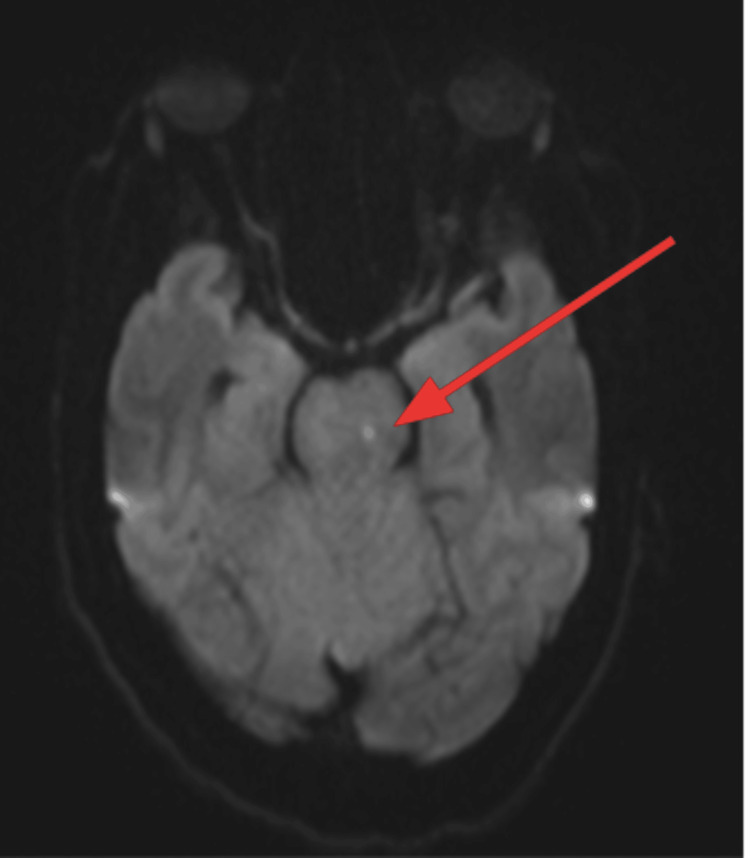
MRI of the brain T2 sequence (axial view) MRI of the brain T2 sequence showed a small hyperintense area in the left pons that correlated with the DWI sequence and ADC map, which was consistent with an acute pontine infarct (red arrow). MRI: Magnetic resonance imaging; DWI: Diffusion-weighted imaging; ADC: Apparent diffusion coefficient

**Figure 4:**
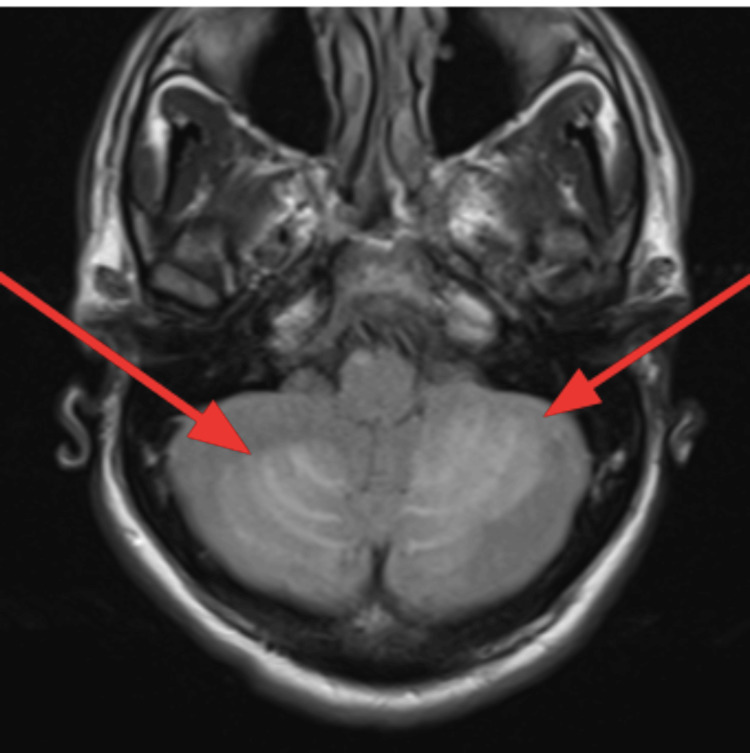
MRI of the brain T2-FLAIR sequence (axial view) MRI of the brain T2-FLAIR sequence showed a large hyperintense area involving the cerebellum, representing cerebellar edema (red arrows). T2 FLAIR: T2-weighted Fluid-Attenuated Inversion Recovery

**Figure 5:**
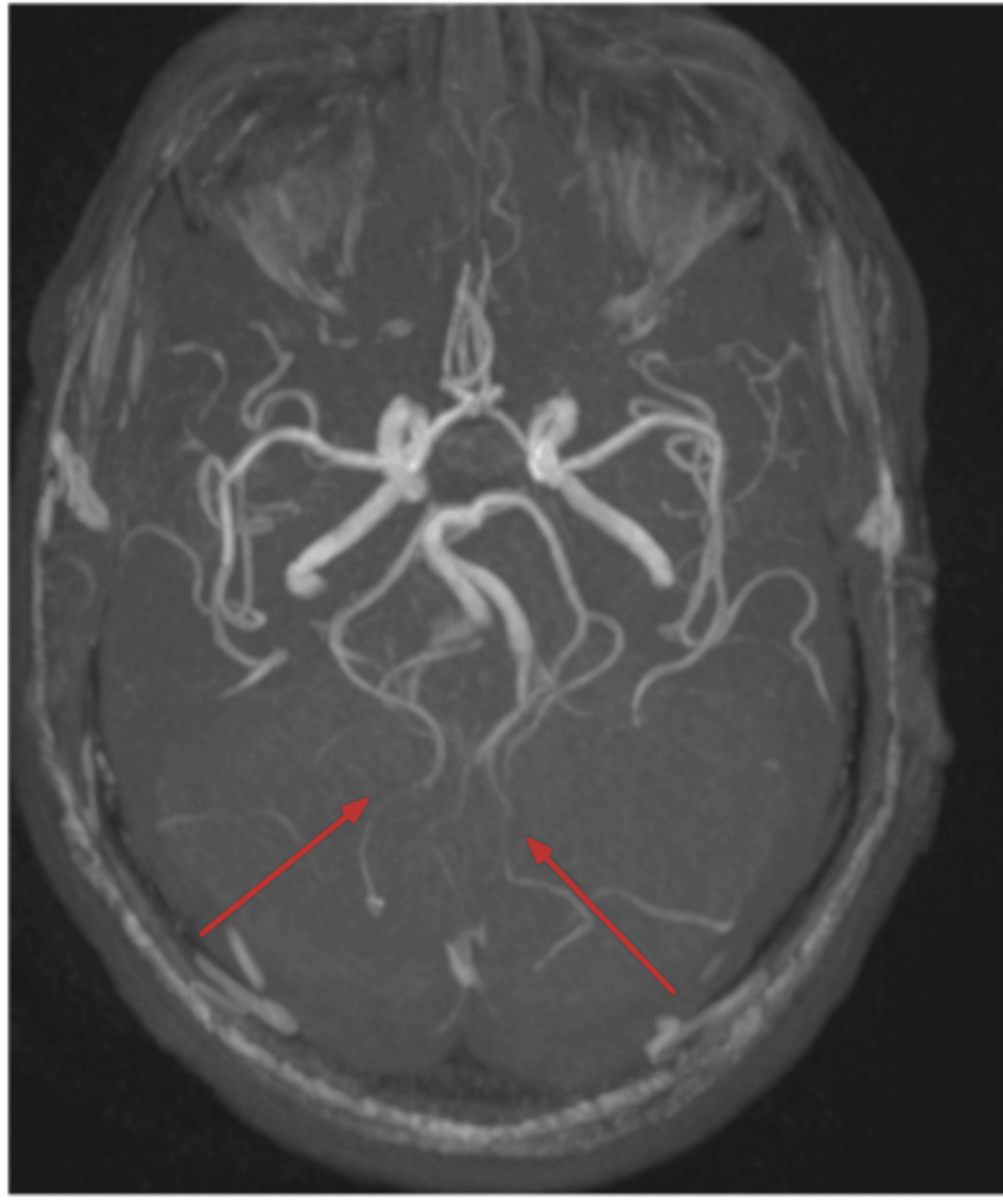
MRA of the brain without contrast (axial view) MRA of the brain revealed a narrowing of blood vessels in the posterior circulation territory representing vasospasm (red arrows). MRA: Magnetic resonance angiography

An EKG was performed, and it showed normal sinus rhythm with left ventricular hypertrophy (Figure 1). A CT of the brain without contrast was performed and showed a large hypodense area involving the cerebellum, representing cerebellar edema and a mild localized mass effect with partial effacement of the fourth ventricle shifted slightly to the right of the midline (Figure 2). An MRI of the brain T2 sequence showed a small hyperintense area in the left pons that correlated with the diffusion-weighted imaging sequence and the apparent diffusion coefficient map, consistent with an acute pontine infarct (Figure 3). A T2-weighted fluid-attenuated inversion recovery (T2-FLAIR) sequence MRI showed a large hyperintense area involving the cerebellum, representing cerebellar edema (Figure 4). A magnetic resonance angiography (MRA) of the brain revealed a narrowing of blood vessels in the posterior circulation territory, representing vasospasm (Figure 5).

A diagnosis of hypertensive emergency was made. He was started on an intravenous nicardipine drip (25 mg + Premix NS 250 mL and intravenous sodium chloride 3% 500 mL) titrated to slowly decrease his blood pressure. The patient's condition improved with the treatment instituted.

**Figure 6:**
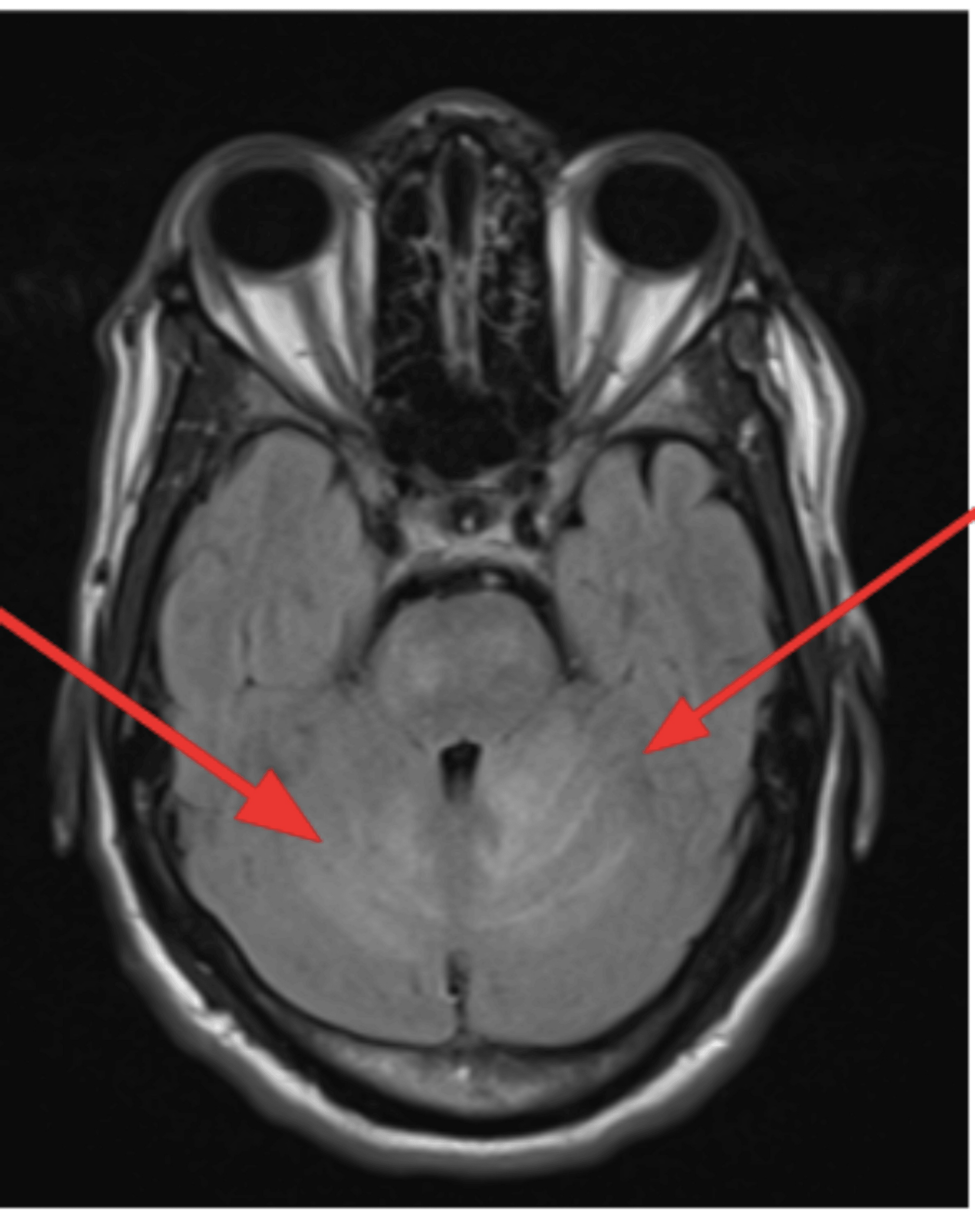
MRI of the brain T2-FLAIR sequence (axial view) MRI of the brain T2-FLAIR sequence showed a decrease in the size of the cerebellar hyperintense area, representing an improvement in cerebellar edema compared with Figure 4 (red arrows). T2-FLAIR: T2-weighted Fluid-Attenuated Inversion Recovery

An MRI of the brain T2-FLAIR sequence was repeated four days after admission and showed a decrease in the size of the cerebellar hyperintense area, representing an improvement in cerebellar edema. (Figure 6).

The patient was discharged five days after admission. He was prescribed aspirin 81 mg, atorvastatin 40 mg, hydralazine 50 mg, labetalol 200 mg, nifedipine 60 mg, and spironolactone 25 mg and advised to follow up with his primary care physician after discharge. 

## Discussion

PRES is a neurologic condition defined by symptoms and imaging findings secondary to vasogenic edema in the brain. According to a Nationwide Inpatient Sample, the prevalence of PRES is 0.03% [4]. It may occur in both sexes and at any age but is more common in middle-aged females, who comprise 63.5% of cases, compared to males, who only account for 36.5% of cases [4]. Etiologies associated with PRES include hypertension, renal disease, preeclampsia, nephrotic syndrome, sepsis, liver disease, and cytotoxic or immunosuppressive medications [1]. Not all hypertensive individuals will progress to PRES, but high blood pressure is the most frequent risk factor associated with the condition. Additional risk factors include autoimmune diseases, seizure disorder, exposure to chemotherapy, and renal failure [1].

The pathophysiology of PRES is not clearly understood, but the most accepted proposed mechanism focuses on the brain's inability to regulate cerebral blood flow through constriction or dilation of vessels during extreme blood pressure [1]. If blood pressure rises above 160mmHg, cerebral vessels constrict to lower pressures and maintain homeostasis, but PRES may occur when these pressure regulatory mechanisms fail, leading to the damage of the blood-brain barrier, extravasation of intravascular fluid, and cerebral edema [1]. Due to fewer pressure regulatory mechanisms, damage to the blood-brain barrier and cerebral edema occurs more frequently in the posterior cerebral circulation than in the anterior cerebral circulation [1]. Criticism of this theory has originated from the fact that PRES has been observed in individuals with blood pressures below the threshold required to trigger regulatory mechanisms of cerebral vessel constriction [1]. Patients with PRES may present with complaints of headaches, altered mental status, seizures, and vision changes such as diplopia, hemianopsia, quadrantanopia, and loss of light perception [1]. Imaging of the brain is required for the diagnosis of PRES. On CT of the head without contrast, brain hypoattenuation indicating vasogenic edema is seen bilaterally in the occipital and parietal regions in a majority of cases [5]. In some patients, hypoattenuation is also seen in watershed areas within the frontal, inferior temporal, cerebellar, and brainstem regions, affecting cortical and subcortical regions [5]. Although a head CT is necessary to rule out emergency causes of seizures, altered mental status, and headaches, an MRI of the brain without intravenous contrast is the preferred diagnostic study to identify vasogenic edema seen in PRES. In an MRI of the brain without contrast, PRES presents as hypointense signals in the affected locations on a T1-weighted MRI and hyperintense signals on a T2-weighted MRI [5]. In a small percentage of cases, a T1-weighted MRI with contrast reveals scattered enhancement in the leptomeningeal or cortical regions. Although unremarkable findings are most common, diffusion-weighted imaging may reveal hyperintense regions or true restricted diffusion [5]. Most cases present with an increased apparent diffusion coefficient, but restricted diffusion has been observed in some patients [5]. An MRA of PRES may reveal signs of vasculopathy and vessel irregularity related to vasoconstriction and vasodilation [5].

A systematic review of 28 studies on PRES found that 11.2% of patients developed an ischemic stroke, 16.1% developed an intracranial hemorrhage, and less frequent complications included a subarachnoid hemorrhage and intraparenchymal hemorrhage [6]. PRES has been found to recur in 4% of patients, especially those with risk factors such as autoimmune diseases, renal failure, and sickle cell crisis [7]. PRES may potentially progress to malignant PRES, which is classified by a Glasgow Score <8, clinical deterioration regardless of interventions to decrease intracranial pressure, and evidence of herniation and mass effect on radiographic imaging [7]. PRES management involves removing the offending trigger, treating the original cause, and cautiously treating hypertension. No standardized antihypertensive treatment has been established, but intervention is indicated if blood pressure measurements rise above 160/110 mmHg [1]. To prevent cerebral ischemia, blood pressure should be reduced by 10-20 mmHg every 10 to 20 minutes [1]. No standard treatment regimen has been verified for seizures in individuals with PRES, but antiepileptic medications may be indicated in the acute phase of PRES [8]. If PRES is related to an immunosuppressive or cytotoxic medication, removal or reduction of the offending medication is indicated. When PRES is associated with a pontine infarct, careful management should be implemented to reduce blood pressure by an estimated 20% in the first few hours [8]. Lowering the blood pressure too excessively can worsen ischemia and lead to cerebral and coronary hypoperfusion [1]. A mean arterial pressure goal of 105-125 mmHg should be obtained before starting long-term aspirin and statin therapy [8].

The prognosis for patients with PRES is largely dependent on the underlying etiology. Mortality has been observed in 19% of cases, and the presence of diabetes mellitus, substantial cerebral edema and hemorrhage on imaging, a delay in the resolution of the etiology, increased C-reactive protein, and involvement of the corpus callosum are associated with negative outcomes in PRES [7]. In a study of cancer patients with PRES, 81% of cases showed resolution on follow-up CT or MRI images, and no deaths were a direct result of PRES [9]. Although the neurological signs and symptoms resolve in most patients after treating the root cause, neurological manifestations may remain in serious cases, including epilepsy, motor deficits, and mydriasis [9].

## Conclusions

Posterior reversible encephalopathy syndrome (PRES) is a neurologic condition defined by symptoms and imaging findings secondary to vasogenic edema in the brain. Severe hypertension is the most frequent risk factor associated with the condition. The pathophysiology of PRES is not clearly understood, but the most accepted proposed mechanism focuses on the brain's inability to regulate cerebral blood flow through constriction or dilation of vessels during extreme blood pressure. It is important to maintain awareness about this condition in all patients who are admitted to the hospital with severe hypertension since its prompt diagnosis and appropriate management are crucial to prevent complications.
